# Methods for the solubilisation of membrane proteins: the micelle-aneous world of membrane protein solubilisation

**DOI:** 10.1042/BST20210181

**Published:** 2021-08-20

**Authors:** Giedre Ratkeviciute, Benjamin F. Cooper, Timothy J. Knowles

**Affiliations:** School of Biosciences, University of Birmingham, Birmingham B15 2TT, U.K.

**Keywords:** membrane protein solubilisation, membrane proteins, membrane solubilisation, smalp

## Abstract

The solubilisation of membrane proteins (MPs) necessitates the overlap of two contradictory events; the extraction of MPs from their native lipid membranes and their subsequent stabilisation in aqueous environments. Whilst the current myriad of membrane mimetic systems provide a range of *modus operandi*, there are no golden rules for selecting the optimal pipeline for solubilisation of a specific MP hence a miscellaneous approach must be employed balancing both solubilisation efficiency and protein stability. In recent years, numerous diverse lipid membrane mimetic systems have been developed, expanding the pool of available solubilisation strategies. This review provides an overview of recent developments in the membrane mimetic field, with particular emphasis placed upon detergents, polymer-based nanodiscs and amphipols, highlighting the latest reagents to enter the toolbox of MP research.

## Introduction

Membrane proteins (MPs) are indispensable components of biological membranes contributing enormously to both their diverse functionality and structure. Intrinsic membrane proteins (IMPs) are integral to the membrane itself, displaying at least one complete traversion of the phospholipid bilayer. Whilst the assumption of the native 3D structure is inherently determined by the amino acid sequence, a synergistic relationship must be established between membrane-spanning residues and lipids to maintain a stable conformation of the correct oligomeric state. However, structural and functional investigations often require extraction of MPs from the heterogeneous membrane and subsequent reconstitution into an environment which permits such downstream analyses.

The act of MP solubilisation is somewhat paradoxical in that it must simultaneously disrupt the native phospholipid environment whilst also stabilising proteins, possessing extensive hydrophobic regions, as they become liberated into an aqueous habitat. The superposition of these two events represents a major challenge in MP structural and functional studies.

**Figure 1.**
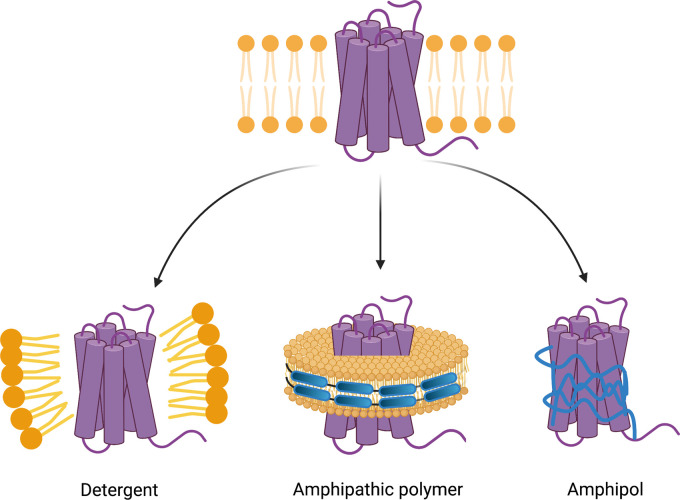
Schematic displaying the extraction of MPs into the three membrane mimetic systems discussed in detail in this review. Left — detergent solubilisation forms a micelle around the hydrophobic region of the MP (purple), middle — solubilisation with a nanodisc forming amphipathic polymer (blue) retains the native membrane lipids (yellow) around the MP, right — amphipols (APols) (blue) self-assemble to mask hydrophobic MP regions from the aqueous environment. Figure created with BioRender.com.

This review aims to provide an overview of the recent innovations in membrane mimetic system innovations, particularly accentuating detergents, polymer-based nanodiscs and amphipols (Figure 1).

## Detergents

The use of detergents, above their critical micelle concentration (CMC), remains the conventional method employed to solubilise MPs prior to biochemical investigation. While the *modus operandi* of the myriad of available detergents is highly related, there are no golden rules for selecting suitable detergents for the solubilisation of a specific MP. Hence, an exhaustive approach must be employed to identify the optimal detergent, for each individual protein, balancing both solubilisation efficacy and protein stability, however, this is rarely achieved owed to the associated cost and time demands, therefore, a traditional detergent satisfying most of the required needs is often implemented instead.

**Figure 2.**
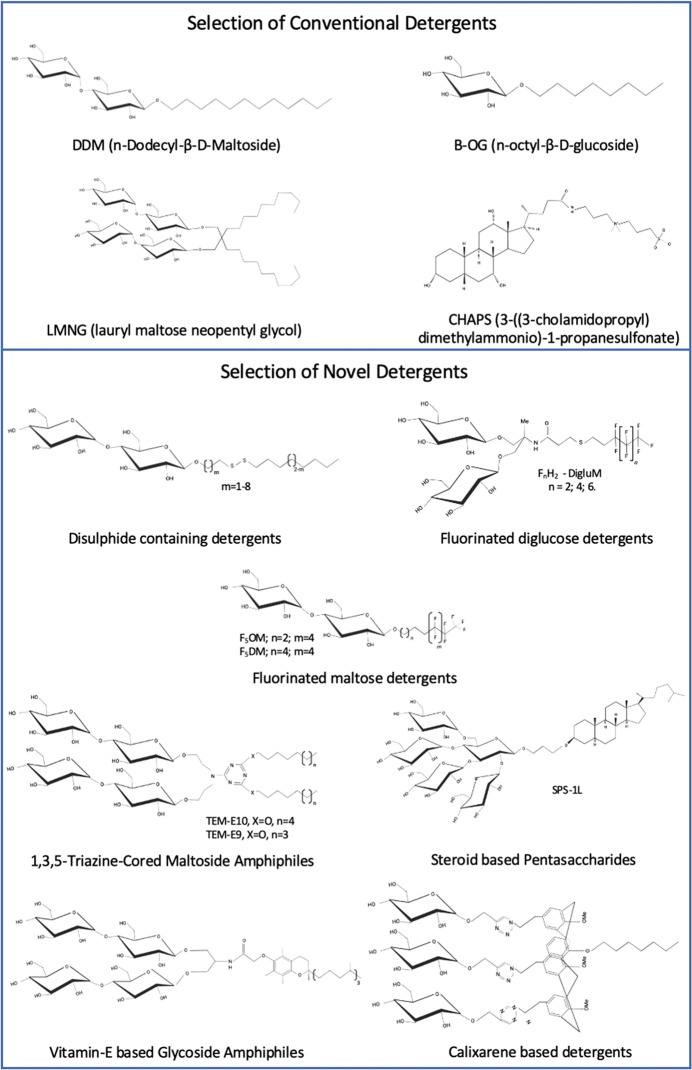
Structures of a selection of detergents referred to within this manuscript. Upper and lower panels depict conventional and novel detergents respectively highlighting the variability within each class and allowing comparison between their architectures.

Conventional detergents, such as n-dodecyl-β-D-maltoside (DDM), are typically comprised of a single alkyl chain and hydrophilic group, with few deviating from this architecture (Figure 2). The limited structural miscellany of conventional detergents is in stark contrast with the far-reaching diversity of MPs hence a ‘one size fits all’ approach to detergent selection appears largely impractical. Indeed, certain MPs undergo degradation or loss of function upon incorporation into micelles of these traditional detergents [1–5], thus the development of novel detergents, which may be employed to study a wider array of MPs, continues to attract significant research effort. Past research has focused primarily upon the evolution of detergent classes, via the introduction and modification of functional groups, to confer improved solubilisation/stabilisation properties. However, recently there has been a drive towards developing completely novel detergents that confer, in particular, elevated protein stability, compared with classical detergents. Examples of these include; a new class of steroid-based pentasaccharides [6], fluorinated glucose and maltose-based detergents [7,8], innovative facial amphiphiles (FAs) [9,10], 1,3,5-triazine-cored detergents [11], disulfide containing amphiphiles [12] and modified neopentyl glycol based detergents that have undergone subsequent modification to further improve their characteristics [13] (Figure 2).

Unique chemical architectures, deviating significantly from those previously developed, have also been proposed. Indeed, five vitamin-E based glycoside amphiphiles (VEGs), consisting of a hydrophobic vitamin E based alpha-tocopherol chain and hydrophilic branched glycoside head group, were recently developed and characterised to assess their potential biochemical application (Figure 2) [6]. Many MPs, including the β_2_AR and its G_s_ complex, displayed elevated stability and retention of functionality over longer incubation periods once incorporated into VEG micelles [6]. Furthermore, negative stain EM of VEG-3 reconstituted β_2_AR–G_s_ complex yielded monodisperse, non-aggregated single particles highlighting a potential application in Cryo-EM [6]. Despite these favourable properties, VEGs presented a solubilisation efficiency of approximately 50–60%, much less compared with the gold standard detergent DDM (90%) [6], therefore, even with this latest generation of detergents, the necessity to extract MPs via conventional detergents remains.

Finally, the calixarene platform, formed by the cyclic oligomerisation of four phenol molecules, has provided a promising backbone for the development of novel detergent architectures. Initial calixarene detergents aimed to increase the stability of extracted MPs via interactions between the calixarene platform and both aromatic residues (π-stacking) and basic residues (salt bridges) often found at the cytosolic-membrane interface [14–18]. Subsequent modifications of the hydrophobic and hydrophilic groups appending the central calixarene platform has yielded an ever-increasing array of calixarene detergents. Of note are modifications to the length of the acyl tail which were shown to dramatically influence solubilisation efficacy. Calixarenes possessing short aliphatic tails, less than 7 carbon atoms in length, displayed poor solubilisation abilities, indeed, compounds with 1 and 3 carbon atom tails failed to extract MPs at all [17]. A distinct improvement in solubilisation efficacy was observed for compounds with a tail ≥7 carbon atoms in length with a 12 carbon tailed derivative extracting BmrA and ABCG2 from insect cells with a greater efficiency than DDM [17]. The correlation between longer alkyl chain detergents and improved extraction of MPs has been shown in several previous studies [19–21]. Detergents with longer chains are inherently more hydrophobic, and thus less soluble, than their short-chain counterparts. Consequently, long-chain detergents possess lower CMC values that enable more effective partitioning of MPs into their larger micelles which feature a greater accessible micellar core volume [21]. After probing the solubilisation characteristics of calixarenes, Matar-Merhab et al. [17], concluded that, despite being anionic in nature, these compounds behave in a similar manner to mild detergents like DDM hence they will likely fail to extract MPs from inclusion bodies or similar materials.

CALX-173-GK, a novel glycosylated calixarene detergent, was recently produced consisting of three saccharide groups, a calixarene ring and hydrophobic tail of seven carbon atoms [22] (Figure 2). While CALX-173-GK shows low solubilisation efficacy, ATPase activity of the BmrA transporter reconstituted in DDM/CALX-173-GK was significantly elevated (more than 6-fold) compared with that reconstituted in DDM alone highlighting the potential advantage of supplementing canonical ‘solubilising’ detergents (DDM) with novel ‘stabilising’ detergents [22]. Recently, Agez et al. [23], demonstrated an alternative approach, deploying two calixarene detergents, CALX-R2 (CALX8) and CALX-173-GK, in tandem to efficiently perform the extraction and stabilisation phases of human CD20 solubilisation respectively considering their differing hydrophobicities and thus ability to partition MPs. Reconstitution into CALX-173-GK micelles resulted in stable, homogenous CD20 particles visible through negative stain EM, which did not fuse or aggregate, highlighting its potential application in Cryo-EM [23].

Despite some of the aforementioned detergents showing superior protein stability, in many cases, the issue of poor solubilisation capabilities remains. Consequently, extraction must typically be performed using conventional detergents prior to exchange into these novel detergents. Such exchange steps may be detrimental to MP structure and impact upon their function [5,24–27]. Such intricacies, coupled with the elevated expense associated with novel detergents mean DDM remains the favourable choice of detergent for MP solubilisation accounting for approximately 50% of all unique structures solved between 2010 and 2019 [28]. Increased research interest in MPs will likely necessitate further assessment and characterisation of such novel detergents, elevating their usage and encouraging further optimisation. The pool of available detergents is becoming ever more diverse thus slowly aligning with the complex nature of MPs.

## Lipid containing systems

Whilst detergent mediated solubilisation of MPs remains the canonical approach, identification of the optimal detergent can be expensive and time consuming, necessitating consideration of numerous variables [29]. Furthermore, micelles present a relatively poor lipid-bilayer mimetic, generally attributed to the lack of lateral pressure usually implemented by the membrane [30]. Furthermore, neutron reflectometry has revealed the membrane bilayer to be a complex structure with physiochemically distinct layers [31], which detergent micelles cannot replicate comprehensively due to their micellar morphology differing fundamentally from the membrane leaflet architecture. Consequently, MP stability, structure and activity within these mimetic systems may be altered compared with those within the native membrane environment.

To alleviate the limitations associated with the structural/function characterisation of detergent-solubilised MPs, detergent-extracted MPs may be reconstituted into systems themselves containing lipids such as mixed micelles, planar bilayers or liposomes. Of the aforementioned systems, liposomes are particularly important owing to their regular employment during functional investigations of MP transporters or indeed the reconstitution of entire transport systems [32–36], and their ability to partition substrates facilitating assaying. However, despite their ability to more accurately resemble the native lipid environment these techniques often yield heterogeneous populations, in terms of size, composition or protein orientation, which hinder further biochemical studies [37]. Furthermore, that MPs must be initially solubilised in detergent, before subsequent exchange into liposomes, raises the possibility of protein inactivation or degradation.

## Nanodiscs

**Figure 3.**
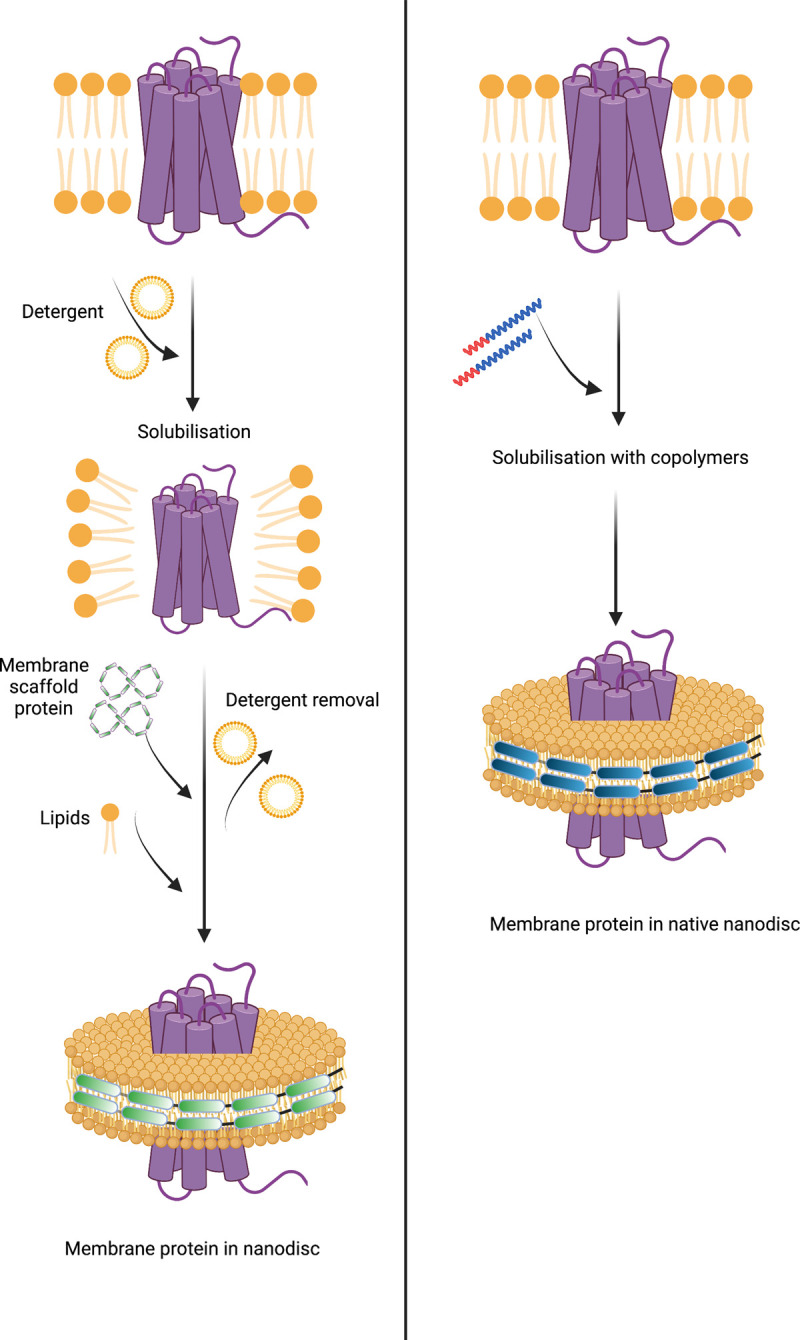
Schematic representation of MP incorporation into MSP and polymer nanodiscs. (Left) MSP nanodisc solubilisation — MP is initially extracted into detergent micelles to which lipid and MSP are added before detergent removal triggers nanodisc formation. (Right) Amphipathic polymer nanodisc solubilisation — addition of amphipathic polymer to the membrane fraction results in the spontaneous formation of nanodiscs encapsulating the native lipid and protein. Figure created with BioRender.com.

Nanodiscs constitute a discoidal phospholipid bilayer section stabilised by either an amphipathic protein, peptide or polymer which act to shroud the hydrophobic phospholipid tails from the aqueous environment. The first nanodisc systems utilised amphipathic, helical belt proteins derived from human ApoA1, the major proteinaceous component of high-density lipoprotein (HDL) particles, to encapsulate the bilayer section (Figure 3) [38,39]. Genetic engineering of ApoA1 yielded an array of so-called ‘membrane scaffold proteins’ (MSPs) enabling modulation of nanodisc size and incorporation of a range of tags [40,41]. Preliminary modifications yielded MSP1, a construct lacking the N-terminal globular domain of ApoA1, and MSP2, a construct containing two copies of MSP1 joined in a head to tail fashion via a short linker [39]. For a comprehensive list of MSP variants developed subsequently, including their sequence composition, please refer to [41].

MSP nanodiscs present an attractive biochemical tool considering their tunable size, lipid composition and tagging capabilities hence their applications are numerous and continue to grow. A sample of MSP nanodisc applications include binding experiments (surface plasmon resonance [42], localised surface plasmon resonance [43]), fractionation methods (electrophoresis [44], ultracentrifugation [45], chromatography [46]), spectroscopic studies [47] and structural determination (NMR [48]), X-ray crystallography [49], electron microscopy [50], small-angle x-ray scattering [51], small-angle neutron scattering [52], analytical ultracentrifugation [53], neutron reflectometry [54] highlighting their diverse potential.

A significant development in nanodisc technology was the utilisation of the styrene maleic acid (SMA) copolymer to form SMA-lipid particles (SMALPs) in 2009 (Table 1) [55]. Such polymer-based nanodiscs display a distinct advantage over their protein stabilised predecessors as they may directly extract proteins from the membrane, negating the need for initial detergent solubilisation, hence lipids contributing to structure or function likely remain associated with the protein following extraction (Figure 3) [56]. To date, SMA solubilisation has been effectively used for a wide range of MPs including ABC-family of transporters [57], ion channels [58,59] and G-protein coupled receptors [60]. However, the incompatibility of SMALPs to low pH (<6.5) and divalent cations (>5 mM) coupled with a tendency to form populations of heterogeneous disc size and interfere with Ni-NTA resin [56,61] severely restricts their usage in structural and functional studies. Consequently, research within the nanodisc field has centred largely upon developing polymers which may be utilised in a wider range of applications than their predecessors as well those possessing novel functionalities.

**Table 1.** Structure of the amphipathic copolymers discussed in this review along with their respective advantages and disadvantages

Polymer	Advantages	Disadvantages
SMA 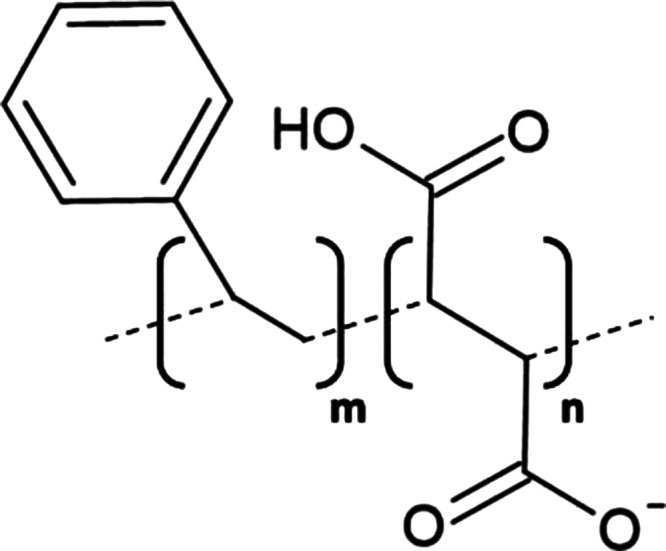	Effective solubilisation of a wide range of MPs Retention of native lipid environment Commercially available	Precipitation at acidic pH (<6.5) Sensitivity to mM concentrations of divalent cations Absorption of UV light (styrene moiety) Nanodisc diameter limited to ∼10 nm May interfere with binding to Ni-NTA resin or other affinity matrices
SMA-ED 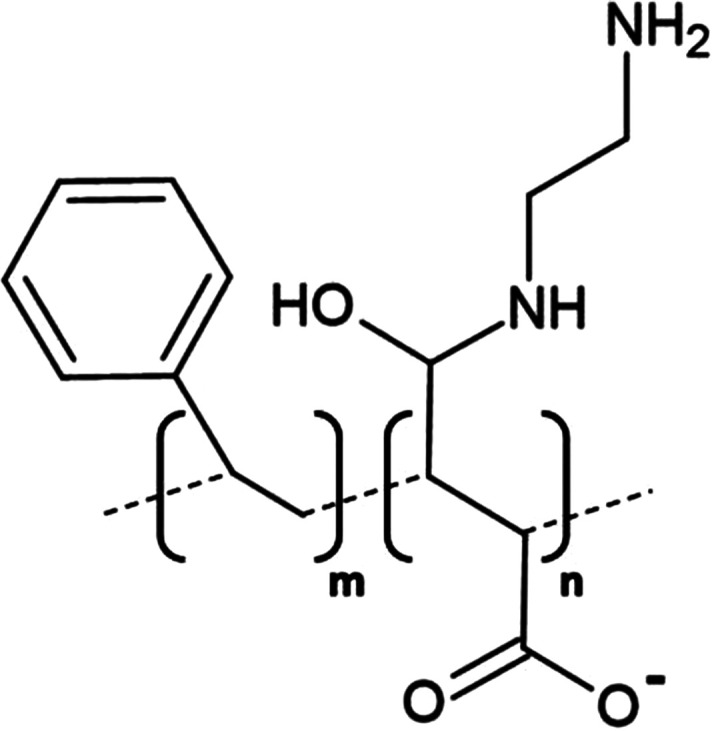	Stability over a greater pH range (pH < 5 and pH > 7) Greater tolerance of salt and divalent cations	Instability between pH 5–7 Absorption of UV light (styrene moiety) Not commercially available
SMAd-A 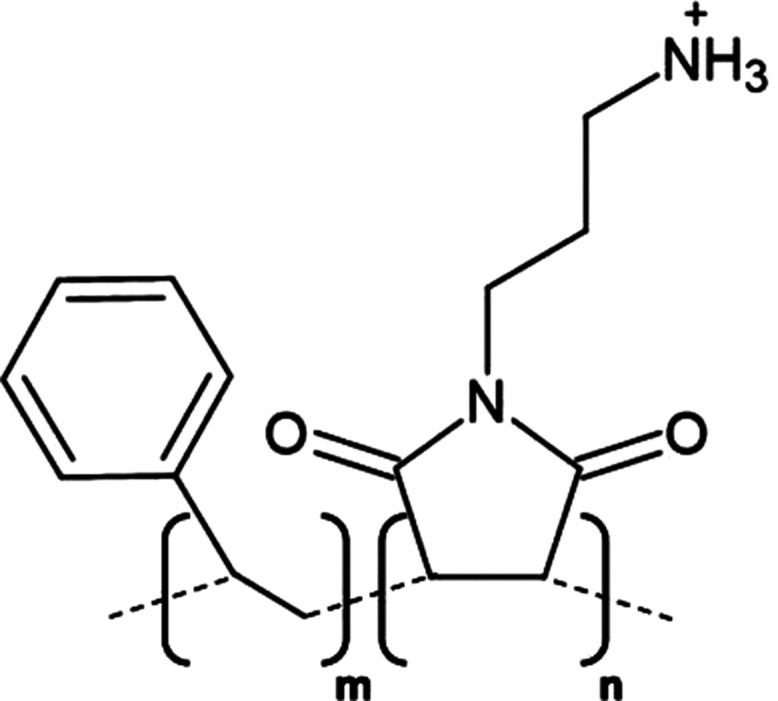	Stability under acidic pH (pH < 6) Greater tolerance of salt and divalent cations	Absorption of UV light (styrene Pmoiety) Precipitation at basic pH (>6) Not commercially available
SMA-QA 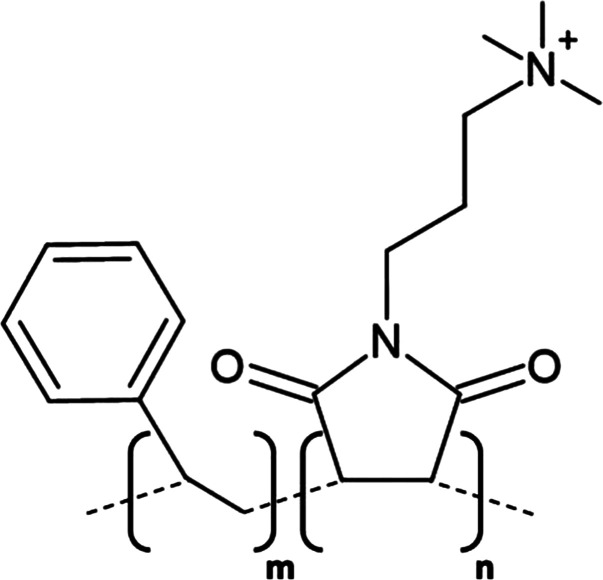	Stable over all biologically relevant pH values (2.5 < pH < 10) Ability to modulate nanodisc size including formation of macro-nanodiscs (∼30 nm) Greater tolerance of salt and divalent cations Alignment in the presence of an external magnetic field	Absorption of UV light (styrene moiety) Not commercially available
SMA-EA 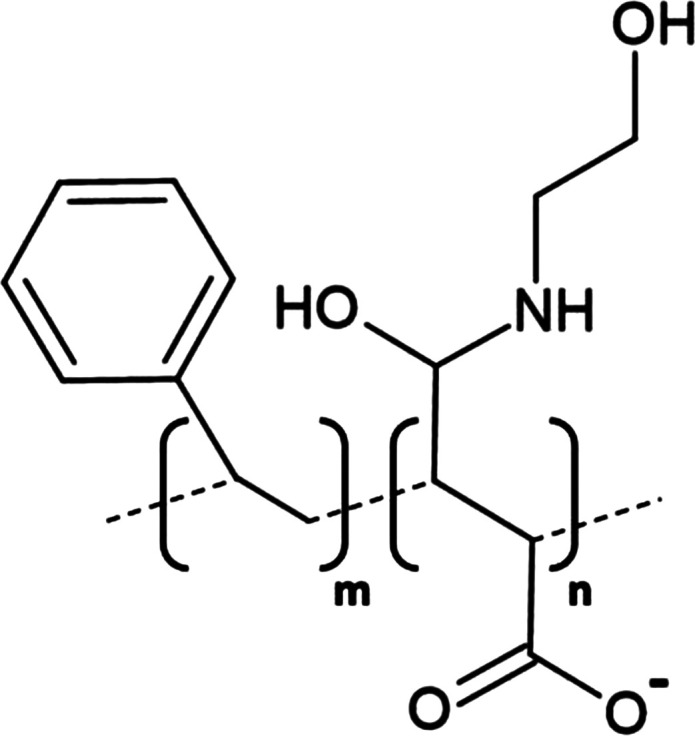	Stable under acidic pH values up to pH ∼3.3 Ability to modulate nanodisc size including formation of macro-nanodiscs (∼60 nm) Greater tolerance of salt and divalent cations Improved thermal stability Alignment in the presence of an external magnetic field	Absorption of UV light (styrene moiety) Not commercially available
SMI 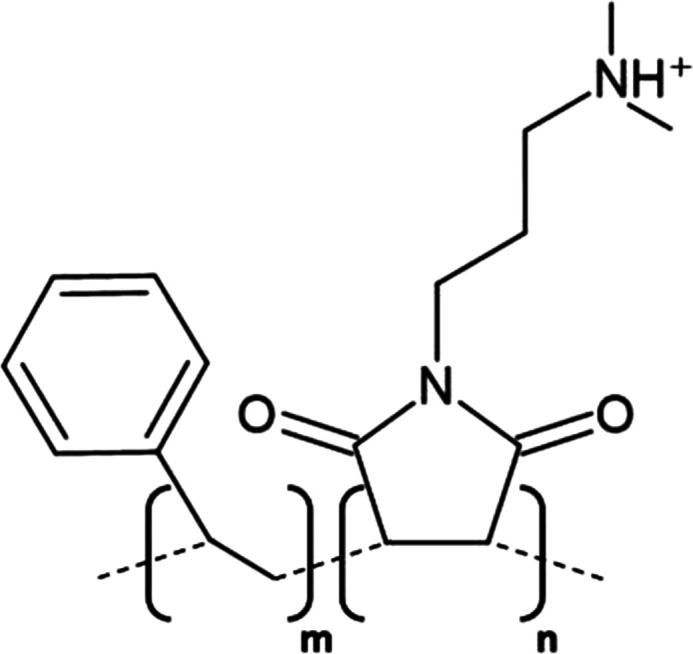	Stable over a greater pH range (pH < 7.8) Completely resistant to divalent cations Smaller nanodisc size Improved thermal stability	Instability at pH > 7.8 Absorption of UV light (styrene moiety) Smaller nanodisc size Possible interactions with biomolecules during the purification process Not commercially available
SMA-SH 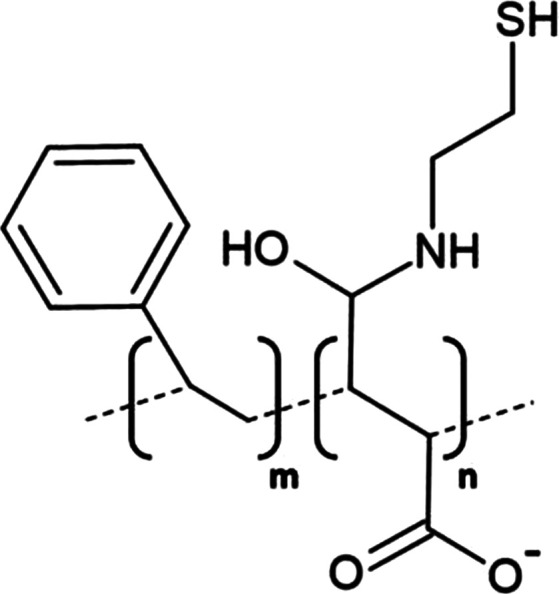	Reactive sulfhydryl group allowing conjugation to thiol-reactive compounds	Precipitation at acidic pH (<6.5) Sensitivity to mM concentrations of divalent cations Absorption of UV light (styrene moiety) Not commercially available
zSMA 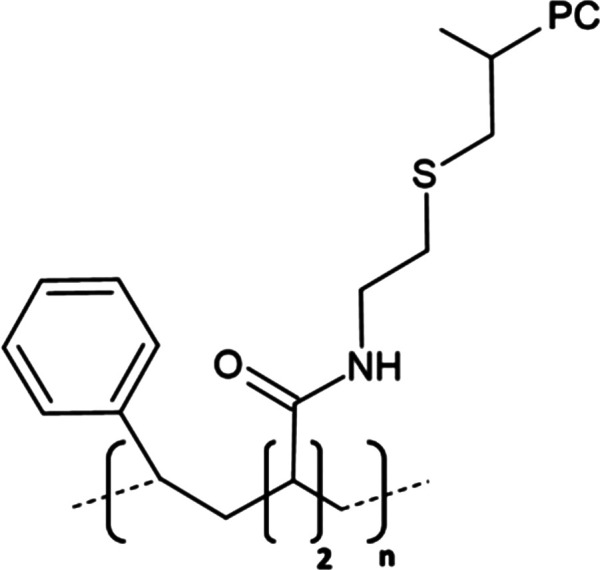	Remains soluble at acidic pH values (down to ∼pH 4) Greater tolerance of salt and divalent cations Modulation of nanodisc size	Absorption of UV light (styrene moiety) Not commercially available
DIBMA 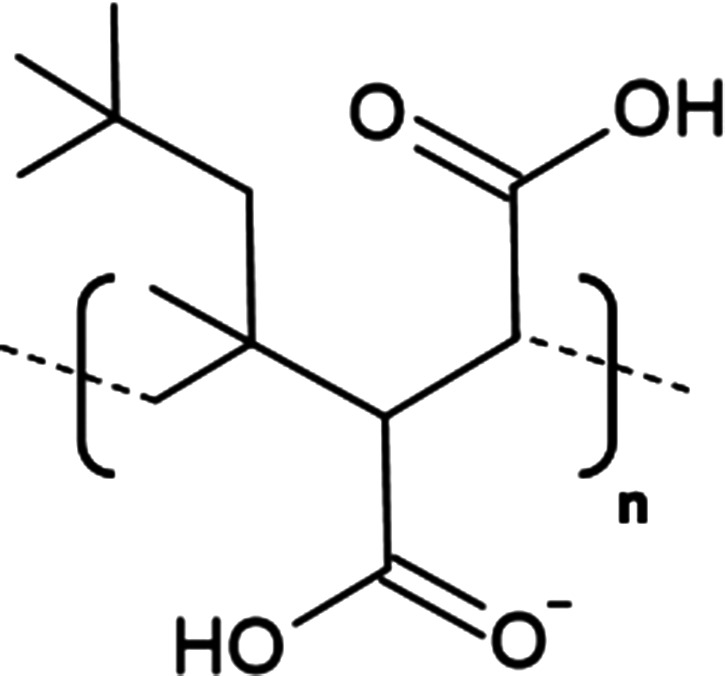	Suitable for UV spectroscopic studies (no styrene moiety) Less perturbation of bilayer dynamics within the nanodisc Greater tolerance of salt and divalent cations Larger nanodisc size (∼20 nm) Commercially available	Precipitation at acidic pH values
PMA 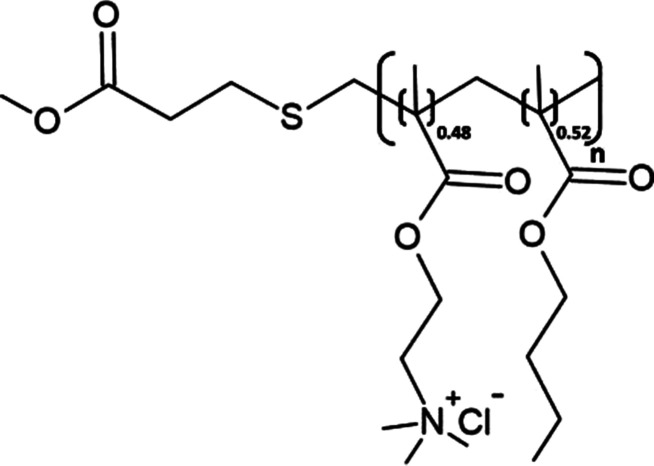	Suitable for UV spectroscopic studies (no styrene moiety) Greater tolerance of salt and divalent cations Effective solubilisation under mild conditions Commercially available	MP solubilisation attributes largely unexplored
Alkyl-PAAs 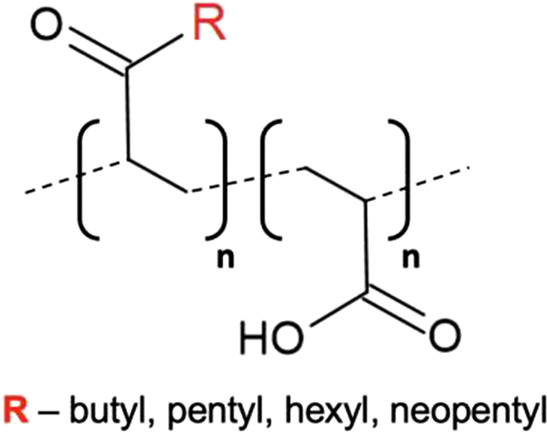	Ability to modulate nanodisc size including formation of macro-nanodiscs Suitable for UV spectroscopic studies (no styrene moiety) Alignment in the presence of an external magnetic field Ability to modulate the degree of bilayer perturbation	Sensitivity to acidic pH and divalent cations MP solubilisation attributes largely unexplored Not commercially available
AASTY 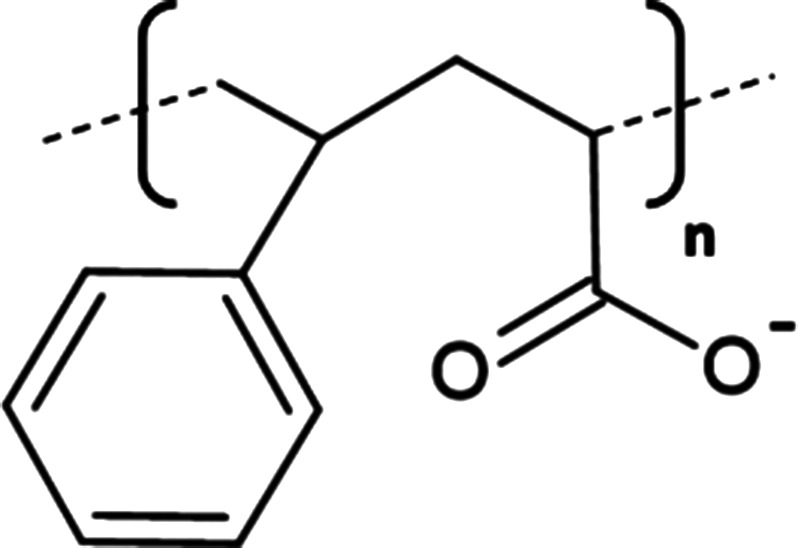	Increased extraction efficacy Greater tolerance of salt and divalent cations	Absorption of UV light (styrene moiety) MP solubilisation attributes largely unexplored Not commercially available

Structures created with ACD/Labs Chemsketch.

Initial modifications entailed variation of the styrene : maleic acid ratio, to adjust polymer hydrophobicity, however, subsequent functionalisation of the maleic anhydride moiety, of a short, commercially available SMA (1.3 : 1) polymer, has conceived an array of polymers with varied characteristics, many of which address the limitations of SMA (Table 1). Functionalisation of SMA with ethylenediamine yields SMA-ED, a zwitterionic polymer, which may be subsequently dehydrated to yield SMAd-A, itself possessing a primary amine [62]. Both of these species display greater tolerance towards salt and divalent cations (up to 200 mM) and are stable under a greater range of pH values [62]. A quaternary amine containing derivative, SMA-QA, is effective over a range of biologically relevant pH values (2.5–10) thus offers a considerable improvement over SMA in this respect [63,64]. In addition, SMA-QA is capable of forming macro-nanodiscs of ∼30 nm in diameter much like SMA-EA, an ethanolamine containing derivative, which may form macro-nanodiscs up to 60 nm in diameter [63–65]. The ability of both derivatives to form a range of disc sizes including macro-nanodiscs, tunable by polymer:lipid ratio, raises the possibility of their utilisation in electron microscopy whilst their magnetic alignment properties may prove beneficial for NMR. A positively charged copolymer, SMI, displays pH stability inverse to that of SMA, remaining soluble only below pH 7.8, is entirely divalent resistant and forms nanodiscs of a smaller diameter, hence it provides an ideal polymer for the solubilisation of membranes and smaller proteins under acidic pHs [66] (Table 1). SMA-SH, an SMA derivative harbouring a sulfhydryl group, may be conjugated to thiol-reactive compounds, or tethered to functionalised surfaces, hence it presents an ideal candidate for surface-based binding assays (surface plasmon resonance, quartz crystal microbalance with dissipation monitoring) and fluorescence studies (Table 1) [67]. Finally, a new family of zwitterionic SMA copolymers (zSMAs) have been developed by substituting the maleic acid moiety of SMA to maleic amide conjugated to phosphatidylcholine groups, with copolymer length defining individual family members [68] (Table 1). zSMA remains soluble at low pH and/or in the presence of millimolar polyvalent cation concentrations whilst crucially polymer size and zSMA nanodisc diameter are positively correlated hence nanodisc size may be easily modulated [68,69].

Another limitation of SMA is its significant absorption of UV light, due to the styrene moiety, which severely interferes with spectroscopic studies of solubilised proteins [70]. To mitigate this issue, polymers lacking the styrene hydrophobic group have been developed. Diisobutylene maleic acid (DIBMA) is composed of alternating diisobutylene and maleic acid moieties [70] (Table 1). DIBMA forms larger nanodics (12–29 nm), with less ordered lipids, potentially providing a more accommodating environment for certain MPs than SMA [70]. In contrast with SMA, DIBMA displays a higher tolerance to divalent cations, enabling its utilisation in biophysical assays which require their presence, such as ATPase activity [70]. Surprisingly, the presence of low millimolar concentrations of Mg^2+^ and Ca^2+^ has been shown to increase the efficacy of DIBMA mediated solubilisation [71]. However, precipitation at acidic pH remains a vulnerability of DIBMA due to retention of the maleic acid carboxyl groups. To date, DIBMA has been successfully implemented in the purification of several MPs, however, when compared directly with SMA, DIBMA nanodiscs typically display reduced stability over time and provide lower protein yields of reduced purity [72].

A poly(methacrylate) (PMA) copolymer was synthesised in 2017 by Yasuhara et al. [73], from hydrophobic butylmethacrylate and cationic methacholine chloride monomers, and has been shown to both solubilise lipid membranes and form nanodiscs (Table 1). PMA is completely styrene and maleic acid free thus this novel polymer provides a promising alternative for biochemical investigation such as circular dichroism or fluorescence-based studies. Despite the anticipated advantages over SMA, only recently have the MP solubilisation attributes of the polymer begun to be assessed. Lavington and Watts [74] successfully performed detergent-free PMA solubilisation of the GPCR neurotensin receptor 1 (NTR1) [74]. PMA displayed solubilisation efficacy comparable to that of conventional detergents in relatively mild solubilisation conditions (26°C pH 7.4–7.6) with no precipitation in the presence of divalent cations thus showing clear advantage over SMA [74]. Such characteristics imply PMA may yet provide a legitimate membrane mimetic for use in biophysical and high-resolution structural studies.

Hydrophobic modification of poly(acrylic acid) (PAA) has previously been shown to yield amphipathic polymers capable of directly extracting MPs from their native membranes [75,76]. Functionalisation of PAA with relatively short alkyl groups (C4–6) has recently been shown to produce a series of amphipathic polymers (alkyl-PAAs) capable of forming nanodiscs, the size of which is dependent upon the lipid : polymer ratio [65]. The tendency of alkyl-PAA macro-nanodiscs to align with a magnetic field suggests a potential application in NMR spectroscopy [65]. Alkyl-PAAs were shown to be capable of extracting MPs directly from the membrane with similar efficacy to SMA [65]. The tolerance of alkyl-PAAs to pH and divalent cations was also shown to be comparable to that of SMA, due to the presence of the carboxylic group, however, the absence of aromatic moieties permits their use in spectroscopic studies [65]. In addition, the length of the functional alkyl chain appeared to directly influence the degree of lipid-bilayer perturbation, with longer alkyl chains generating increased disorder of the encased lipids, suggesting simple modifications may enable modulation of alkyl-PAA solubilisation characteristics [65].

PAA also provided part of the basis for the recently developed poly(acrylic acid-co-styrene) copolymer (AASTY) which comprises styrene and acrylic acid moieties [77]. AASTY has been shown to efficiently extract hTRPM4 from mammalian cells into nanodiscs, with two of the four AASTY polymers assessed displaying extraction efficacies approximately five times that of SMA2000, and display increased stability in the presence of divalent cations [77]. Single-particle cyro-EM of AASTY solubilised hTRPM4 appeared to show promising results, however, the sample exhibited insufficient homogeneity for a high-resolution structure, although this may in part be due to intricacies associated with the specific protein utilised in this study rather than the polymer itself [77]. Considering the favourable aforementioned characteristics of alkyl-PAAs and AASTY, PAA presents a promising foundation for the development of future polymer architectures.

## Amphipols

Amphipols (APols) are short amphipathic polymers that self-assemble to conceal hydrophobic MP portions, maintaining their solubility in aqueous solutions, developed to address detergent-associated stumbling blocks in MP research [78] (for comprehensive reviews please refer to [1,79]). APols themselves do not typically solubilise biological membranes [80,81], thus extraction of MPs must be performed using conventional detergents, however, that APols are very weak detergents may facilitate MP stabilisation. APols do not compete efficiently with protein/lipid interactions thus, dissociated lipids are permitted to rebind upon detergent-Apol exchange, enabling MPs to subsist in a more native, stable state.

The most characterised and widely utilised APol to date is polyacrylate-based A8–35 that has been applied in the study of several MPs including bacteriorhodopsin [82] and cytochrome b_6_f [3]. Despite multiple favourable characteristics, an apparent sensitivity to pH [76] and divalent cations [83] necessitated the development of A8–35 derivatives with modified structures, examples include; non-ionic glycosylated APols (NAPols) [80,84,85], sulfonated APols (SAPols) [86] and phosphorylcholine-based APols (PC-APols) [87]. In addition, APols have been labelled/functionalised to expand their use in MP characterisation including isotopic labelling, the addition of affinity tags and fluorophores (Extensively reviewed in [88]). More recently, Bosco et al. [89], developed biotin functionalised non-ionic APols (BNAPols) that successfully stabilised immobilised functional growth hormone secretagogue receptor (GHSR) upon a streptavidin-coated surface enabling screening of potential ligands via a binding assay.

For single-particle Cryo-EM APols provide an attractive proposition as they eliminate the background noise, often present due to free micelles in detergent-protein samples, and potentially enable proteins to retain co-purifying lipids that may lock them in more stable and or native conformations. APol A8-35 and PMAL-C8 are the most commonly used for determining high-resolution structures with the highest achieved being the bovine-bestrophin-2 anion channel at 2.17 Å [90]. The aptness of APols for Cryo-EM stimulated the development of novel CyclApols which consolidate the properties of SMA and A8–35. Replacing the linear n-alkyl chain of classical A8–35 with cyclic hydrocarbon groups confers the ability of CyclAPols to efficiently solubilise biological membranes [91]. Mimicking this property of SMA eliminates the requirement for classical detergent mediated extraction and subsequent APol exchange that can, in some cases, result in MP destabilisation. A recent preprint reported the use of CyclAPols in the solubiliation of the model bacterial membrane transporter AcrB which subsequently yielded a 3.2 Å Cryo-EM structure [92].

## Conclusion

Artificial systems that conceal and stabilise the hydrophobic regions of MPs, allowing their characterisation in aqueous environments, are only epigones of the native lipid environment. Whilst the diversification of MPs is of paramount importance to cellular processes, it also means that the process of liberating and incorporating individual MPs into membrane mimetic systems, without compromising the native structure and/or function, is highly MP-specific. Hence, numerous time consuming, expensive considerations and screening steps are typically employed prior to successfully obtaining a MP isolated in a state suitable for structural/functional investigations.

In the past decade, numerous diverse lipid membrane mimetic systems have been developed, expanding the pool of available solubilisation strategies. Canonical detergents have been further modified, giving rise to an array of innovative structures displaying improved MP stabilisation characteristics. In addition to SMA and its subsequent derivatives, which display increased tolerance to cations and acidic pHs, novel polymers with distinct architectures have been developed. Despite showing initial favourable properties, these polymers require further characterisation to comprehensively assess the appropriateness of their utilisation in MP solubilisation. Finally, a new class CyclAPols, capable of directly extracting MPs from the membrane and maintaining their solubility in aqueous environments, appear a promising candidate for Cryo-EM applications.

Presently, conventional detergents remain the forerunners in MP research owed to their well-defined characteristics, cost and an extensive repertoire of successful applications encompassing high-resolution structures and functional characterisation. However, it remains unequivocal that the ongoing development of membrane mimetic systems is of paramount importance to facilitate future MP characterisation.

## Perspectives

- *Highlight the importance of the field*: MP are indispensable components of biological membranes, however, their structural and functional investigation often necessitates their extraction from the heterogeneous membrane environment into a more consistent background. The development of novel and the evolution of existing membrane mimetic systems is, therefore, essential to expand the repertoire of MPs available for scientific and pharmaceutical research.
- *Summary of the current thinking*: The array of membrane mimetic systems available to researchers continues to grow, now encompassing three major categories: Detergents, Nanodiscs and Amphipols. However, solubilisation of most MPs continues to be performed by canonical detergents, particularly maltosides such as DDM, with the more novel systems typically reserved for niche cases, often to accommodate a specific technique, or upon exhaustion of traditional detergent options.
- *Comment on future directions*: The plethora of existing membrane mimetics will likely adequately service the extraction and stabilisation of most MPs, however, those requiring more specialist mimetics will likely increase as research interest focuses upon more complex MPs and their complexes. Increased utilisation of these more novel mimetics, and those yet to be conceived, will assist in their optimisation, characterisation and may result in their adoption as more mainstream techniques. However, it must be acknowledged that classical detergents, particularly maltosides, will likely remain at the forefront of MP solubilisation.

## Open Access

Open access for this article was enabled by the participation of University of Birmingham in an all-inclusive *Read & Publish* pilot with Portland Press and the Biochemical Society under a transformative agreement with JISC.

## Abbreviations

AASTY: poly(acrylic acid-co-styrene) CoPolymer
APols: amphipols
B_2_AR: B_2_ adrenergic receptor
BNAPols: biotin functionalised non-ionic amphipols
CMC: critical micelle concentration
Cryo-EM: cryo-electron microscopy
CyclApols: amphipols bearing cycloalkane side groups
DDM: n-dodecyl-β-d-maltoside
DIBMA: diisobutylene maleic acid copolymer
Fas: facial amphiphiles
GHSR: growth hormone secretagogue receptor
GPCR: G-protein coupled receptor
HDL: high-density lipoprotein
LSPR: localised surface plasmon resonance
MP: membrane protein
MSP1D1: membrane scaffold protein 1D1
MSPs: membrane scaffold proteins
NAPols: non-ionic glycosylated amphipols
NMR: nuclear magnetic resonance
NTR1: neurotensin receptor 1
PAA: poly(acrylic acid)
PC-APols: phosphorylcholine-based amphipols
PMA: poly(methacrylate)
SAPols: sulfonated amphipols
SMA: styrene maleic acid copolymer
SMALP: styrene maleic acid lipid particles
VEG: vitamin-E based glycoside amphiphiles
zSMAs: zwitterionic SMA
